# Potentially burdensome care at the end-of-life for people who had a death from cancer: A retrospective population-level cohort study

**DOI:** 10.23889/ijpds.v9i5.2480

**Published:** 2024-09-18

**Authors:** Rebecca Mitchell, Nicholas Burns, Nicholas Glozier, Olav Nielssen

**Affiliations:** 1 Australian Institute of Health Innovation, Macquarie University; 2 Justice Health and Forensic Mental Health Network; 3 Bloomfield Hospital; 4 Faculty of Medicine and Health, University of Sydney

## Objectives

To examine factors associated with indicators of potentially burdensome care provided in hospital, and use of hospital services in the last 12 months of life for people who had a death from cancer.

## Approach

A population-based retrospective cohort study of people aged ≥20 years with a cancer-related cause of death during 2014-2019 in New South Wales, Australia using linked hospital, cancer registry and mortality records. Ten indicators of potentially burdensome care were examined.  Multinominal logistic regression examined predictors of a composite measure of potentially burdensome care, derived by summing 4 indicators: >1 ED presentation; >1 hospital admission; ≥1 ICU admission within 30 days of death; died in acute care (scored as 0, 1 ≥2).

## Results

Of the 80,005 cancer-related deaths, 69.1% had none, 20.0% had 1 indicator, and 10.9% had ≥2 indicators of potentially burdensome care.  Compared to having no indicators of potentially burdensome care, people who smoked, lived in rural areas, were most socially economically disadvantaged, and had their last admission in a private hospital were more likely to experience potentially burdensome care. Older people (≥55 years), females, people with 1 or ≥2 Charlson comorbidities, people with neurological cancers, and people who died in 2018-2019 were less likely to experience potentially burdensome care.

## Conclusions and implications

This study shows the challenge of delivering health services at end-of-life.  Opportunities to address potentially burdensome EOLC could involve taking a person-centric approach to integrate oncology and palliative care around individual needs and preferences.

